# The Gambler’s Fallacy Is Associated with Weak Affective Decision Making but Strong Cognitive Ability

**DOI:** 10.1371/journal.pone.0047019

**Published:** 2012-10-05

**Authors:** Gui Xue, Qinghua He, Xuemei Lei, Chunhui Chen, Yuyun Liu, Chuansheng Chen, Zhong-Lin Lu, Qi Dong, Antoine Bechara

**Affiliations:** 1 National Key Laboratory of Cognitive Neuroscience and Learning, Beijing Normal University, Beijing, China; 2 Department of Psychology, University of Southern California, Los Angeles, California, United States of America; 3 Department of Psychology and Social Behavior, University of California Irvine, Irvine, California, United States of America; 4 Center for Cognitive and Behavioral Brain Imaging and Department of Psychology, Ohio State University, Columbus, Ohio, United States of America; Centre national de la recherche scientifique, France

## Abstract

Humans demonstrate an inherent bias towards making maladaptive decisions, as shown by a phenomenon known as the gambler’s fallacy (GF). The GF has been traditionally considered as a heuristic bias supported by the fast and automatic intuition system, which can be overcome by the reasoning system. The present study examined an intriguing hypothesis, based on emerging evidence from neuroscience research, that the GF might be attributed to a weak affective but strong cognitive decision making mechanism. With data from a large sample of college students, we found that individuals’ use of the GF strategy was positively correlated with their general intelligence and executive function, such as working memory and conflict resolution, but negatively correlated with their affective decision making capacities, as measured by the Iowa Gambling Task. Our result provides a novel insight into the mechanisms underlying the GF, which highlights the significant role of affective mechanisms in adaptive decision-making.

## Introduction

Humans often make non-optimal decisions involving random events. One such example is the gambler’s fallacy (GF), which is the belief that the occurrence of a certain random event is less likely after a series of the same event. The GF has been found to bias individuals’ judgments and decisions in many situations, such as gambling [1], lottery play [2], stock investment [3], and many laboratory tasks [4]. One typical pattern of the decisions guided by the GF is that people are more likely to predict the break of a streak when the streak gets longer.

The GF has been traditionally considered as a heuristic bias characterized by the law of small numbers [5], [6], that is, a segment of a random sequence should reflect the overall distribution. Although the heuristic bias hypothesis suggests that the GF is supported by a fast, emotional and intuitive system, and that it can be overcome by deliberative reasoning [7], [8], emerging evidence, mainly from neuroscience studies, implicates that the GF might result from imbalanced cognitive and emotional decision making mechanisms [4], [9]. In particular, we have formulated the hypothesis that the GF is associated with (1) weak function in the affective decision making system, and (2) strong function in the cognitive system supported by the lateral prefrontal cortex (LPFC) [10].

Abundant evidence from animal research, lesion patient studies and functional imaging studies have emphasized the role of the LPFC in general intelligence and top-down executive controls [11], [12], [13], [14], [15], [16]. In terms of decision making, the LPFC plays an important role in detecting and constructing patterns [17] and updating decision-making strategy according to context [18], which are two component processes required to implement the GF strategy. The GF-like decision (e.g., more risk-taking behavior after losses than after wins) was correlated with left prefrontal cortex activity [10]. A recent study used a combination of functional magnetic resonance imaging (fMRI) and transcranial direct current stimulation (tDCS) technologies in an attempt to establish a causal relationship between activities in the prefrontal cortex and the use of the GF strategy [4]. The results suggest that brain responses in the left lateral prefrontal cortex (LPFC) to the current outcome preceded the use of the GF strategy that followed 10 seconds later. Furthermore, anodal tDCS over the left LPFC, which enhanced the LPFC function, increased the use of the GF strategy.

On the other hand, the GF could be further increased by a weak affective decision making mechanism. Patients with impaired affective decision making due to lesions in the mesial OFC/ventromedial prefrontal cortex and the amygdala exhibited behavioral patterns that assemble the GF [9], [19]. For example, in the Iowa gambling task (IGT) that simulates daily-life decision-making [9], healthy participants gradually shift to advantageous decks by (implicitly) developing predictive somatic responses to disadvantageous decks, whereas patients with focal brain damages in the ventromedial PFC keep choosing the disadvantageous decks after severe losses [9], [20]. The patients showed normal switching to other decks after receiving a large loss, but just returned to bad deck (e.g., Deck B) more quickly than the healthy controls. When the patients were confronted with the question: ‘‘Why are you selecting the decks that you have just told me were bad decks?’’ the most frequent answer has been ‘‘I thought that my luck is going to change’’ (unpublished clinical observations). Similarly, in an investment game [19], the VMPFC and amygdala patients with impaired affective decision making (as measured by the IGT) continued to invest after several losses, providing preliminary evidence for the association between VMPFC function, affective decision making and the GF. For healthy subjects, the VMPFC also showed reduced activity to gains than to losses under long streaks [4], which presumably also facilitated the implementation of the GF strategy under long streaks.

With behavioral data from a large sample of 438 college students, the present study aimed at examining both hypotheses on the same subject population using an individual differences approach. We predicted that subjects with strong cognitive ability, as reflected by higher general intelligence and executive function, such as working memory and conflict resolution, would show more GF. In contrast, subjects with stronger affective decision-making capacity, as measured by the IGT, would show less GF.

## Methods

### Participants

Four hundred and thirty-eight (234 females, 20.5±0.98 years old) Chinese undergraduate students volunteered to participate in this study. They were a subsample of a large-scale gene-brain-behavior project (see [21] for more information). They had normal or corrected-to-normal vision, and had not had neurological or psychiatric problems. Informed written consents were obtained from all participants and the study was approved by the Institutional Review Board of the State Key Laboratory of Cognitive Neuroscience and Learning at Beijing Normal University.

### The Gambler’s Fallacy Task

The GF was measured by a matching-pennies game implemented in a Card Guessing Task [4], where subjects were asked to guess the computer’s choice of red or black cards in order to win money. They won one Chinese Yuan (RMB) each time they guessed correctly but otherwise lost one Yuan. They were told explicitly that the computer chose the card *randomly*. Each trial lasted 6 seconds: First, two cards (red and black) were presented on the left and right sides of the screen, respectively. The position of the red or black card varied randomly from trial to trial to dissociate decision switch from motor switch. The computer made the choice after one second. The subjects were then asked to make a guess within 2 seconds. Half second after the subjects’ choice, both the computer and subjects’ choices were revealed and feedback was delivered for one second. The next trial started 2 seconds later. To reduce the short-term memory load, the computer’s last five choices were presented on the top of the screen. Subjects finished two sessions of the computer task containing 63 trials.

Essential to the task, the computer’s choices followed a predetermined, canonical random sequence generated by a Bernoulli process characterized by (1) equal number of black and red cards, (2) switch of card choice on half of the trials, and (3) streak length in an exponential distribution. The procedure guarantees that at any streak length, the probability that a streak will continue or break is always 50%. The optimal strategy (i.e., Nash equilibrium) is to choose the red or black card randomly.

A subject using the GF strategy would predict that the computer’s choice is more likely to switch in the next trial when the streak gets longer. Thus, the GF is defined as a strategy to *deviate* from the computer’s last choice, which can be used under both short and long streaks. This is in contrast to the win-stay-loss-shift (WSLS) strategy in stochastic decision making, which refers to the strategy to *follow* the computer’s last choice [18]. That is, with the WSLS strategy, if the player's choice matches the computer’s choice in this trial (win), he or she will stick to this (also the computer’s) choice in the next trial (stay); otherwise (loss), he or she will switch (shift) to the other choice, which is the computer’s current choice.

### Tasks to Measure Intelligence and Executive Function

The standard Raven’s Advanced Progressive Matrices (RAPM) and Wechsler Adult Intelligence Scale-Revised Chinese Version (WAIS-RC) were used to measure general intelligence, and detailed description can be found in our previous work [21], [22]. The number of correct responses to the test items of RAPM and two IQ scores (verbal IQ, performance IQ) of WAIS-RC were used to index intelligence for this study.

The executive function was tested with a 2-back working memory task (WMT) [21] and the Stroop task [23]. Briefly, in the WMT, subjects were asked to perform a 2-back task based on the semantic (i.e., semantic category) and phonological (rhyme) information on Chinese characters, and the morphemic information (whether two characters were the same) on unfamiliar Tibetan letters. The average score (accuracy) of three tasks was used as the index of working memory performance. Cronbach alpha in this sample was.82.

For the Stroop task, the classic Color-Word Stroop task with manual response was used. Four colors (Red, Green, Blue and Yellow) and corresponding words were used to generate congruent and incongruent trials. Participants were asked to respond to the printed color (not the meaning of the word) using four buttons as quickly as possible. The reaction time difference between incongruent and congruent trials was taken as the measure of conflict resolution aspect of executive function.

**Figure 1:**
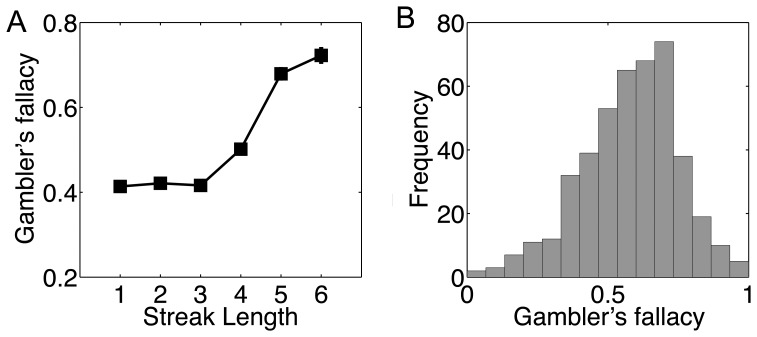
Behavioral performance in the gambler’s fallacy task. A. Percentage of trials using the gambler’s fallacy strategy (i.e., deviating from computer’s last choice) as a function of streak length. Error bars (which is very small and invisible except streak 6) represent standard errors. B. Histogram of individual differences in the use of the gambler’s fallacy strategy under long streak (> = 4).

**Table 1:** Descriptions of major measures.

Domain	Task	Measure	N	Mean	SD	Min	Max
**Gambler’s fallacy**	GF	GF (%)	438	58.40	18.46	0	100
**Intelligence**	RAPM	RAPM test score	434	25.67	4.03	12	35
	WAIS	Verbal IQ	432	123.82	8.69	97	143
		Performance IQ	432	123.50	9.58	95	147
**Executive function**	Stroop	RT (ms): Incong – Cong^a^	411	137.48	72.66	1.69	345.59
	WMT	Accuracy (%)	420	85.90	6.69	61	98
**Decision making**	IGT	(C+D)–(A+B): first 40 trials	422	−5.21	10.19	−38	28
		(C+D)–(A+B): last 60 trials	422	10.73	25.72	−54	60

a Cong: Congruent; Incong: Incongruent.

b RA: Risk Advantageous; RD: Risk Disadvantageous.

### Tasks to Measure the Affective Decision Making Capacity

The Iowa Gambling Task (IGT) [9] was used to measure the affective decision making capacity. A detailed description of the IGT used in this study can be found in He et al. [21]. Previous study has shown that on average, decisions in the first 40 trials were made under ambiguous conditions, but after that, subjects began to develop some subjective sense of the probabilities and thereby began to make decisions under risk [24]. This distinction has been widely adopted by later studies [25], [26]. Genetic [21] and pharmacological studies [27] have also suggested that performances in the two stages may be affected by different neural transmitters and are subject to different genetic influence. Following these studies, the IGT scores (good – bad decks) for the first 40 trials as well as for the last 60 trials were calculated, representing affective decision making under ambiguity and risk, respectively.

**Table 2:** Correlations between different measures.

	RAPM	Verbal IQ	Perf IQ	Stroop	WMT	IGT First40
Verbal IQ	.184**					
Perf IQ	.428**	.283**				
Stroop	−.087	−.034	−.137**			
WMT	.217**	.154**	.210**	−.160**		
IGT First40	−.006	−.077	−.032	.025	−.023	
IGT Last60	.071	.007	.136**	.034	.005	.327**

Note: p<.05;

** p<.01.

**Table 3:** Component matrix for behavior measures.

	Cognitive ability	Affective decision making ability
Perf IQ	**.775**	.164
RAPM	**.716**	.123
WMT	**.547**	**−**.033
Verbal IQ	**.532**	**−**.160
Stroop	**−.370**	.078
IGT First40	.081	**.809**
IGT Last60	**−**.123	**.798**

Extraction method: Principle component analysis. Rotation method: Varimax with Kaiser Normalization.

## Results

Table 1 showed descriptive statistics (N, mean, SD and Range) for the main measures. Note that due to technical errors, not all subjects had data for all the tasks. Focusing on the GF task, we found that, under short streaks (i.e., 1–3), subjects mainly used the WSLS strategy, but they shifted to the GF strategy starting with a streak length of 4 (Figure 1A), consistent with many previous observations [3], [4], [28]. We thus calculated the GF strategy rate only under the long streaks (> = 4) and use it as a measure of individuals’ GF strategy tendency. The averaged GF rate under long streaks for our highly educated and intelligent (average IQ, 124±9 as measured by both RAMP and WAIS) college students is 58.40%, which is significantly higher than 50% (p<.001). More importantly, we found that the range of GF rate varied from 0% to 100%, suggesting significant individual differences (Figure 1B).

As shown in Table 2, the different measures of cognitive ability were correlated with each other, but not with the measures of affective decision making. Exploratory factor analysis was then performed on these measures. Kaiser-Meyer-Olkin (KMO) test measure of sampling adequacy (KMO = .54) and Bartlett’s test of sphericity (χ^2^(21) = 212.76, p<.001) suggested our data was suitable for factor analysis. Principle component analysis (PCA) extracted two components with factor eigenvalue greater than 1, which in total explained 45.54% of the variance. Varimax with Kaiser Normalization was used to rotate the loading matrix. As shown in Table 3, the RAPM, WAIS-RC, WMT and Stroop tests primarily loaded on the first factor, which was named the cognitive ability; in contrast, the two IGT scores primarily loaded on the second factor, which was named the affective decision making ability.

Next we examined the core hypothesis of this study by correlating individuals’ GF strategy use with their cognitive and affective decision making capacities. Consistent with our hypothesis, we found that subjects’ GF strategy use was positively correlated with the cognitive ability (r = .170, p<.001), suggesting that higher intelligence and higher executive function was associated more GF strategy use. In contrast, we found that the use of GF strategy was negatively correlated with affective decision making ability (r = −.109, p<.033), suggesting that subjects with higher affective decision-making ability showed lower GF rate in the task.

**Figure 2:**
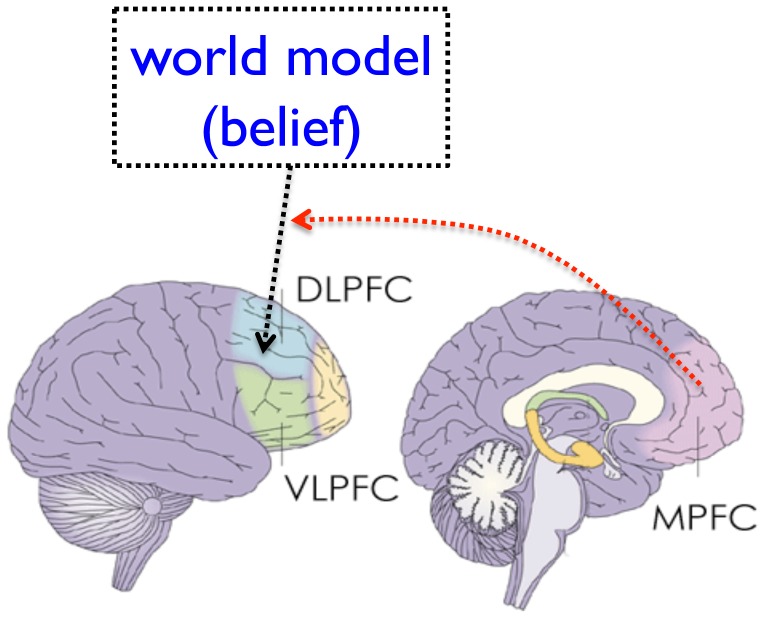
A proposed three-component model for model-based decision making, including an abstract world-model, the LPFC cognitive system and the MPFC affective system. Depending on the situations and the subjective world model, the LPFC and the MPFC could both play constructive and/or destructive roles.

## Discussion

Many early behavioral studies have suggested that the gambler’s fallacy (GF) is determined by the wrong beliefs (or world models) that are probably formed through years of evolution in response to the real world. In addition to the law of small numbers [5], other beliefs regarding the randomness of underlying generating mechanism [28], [29], the “gestalt” of the events [30], the replenishment of natural reinforcers [31], could all affect the GF strategy. Computational models show that a rational mind guided by a false “world model” (i.e., outcome dependency) could well generate this type of suboptimal decisions, which can be changed by alternations of the “world model” [32].

Although many previous studies suggest higher cognitive skills are usually associated with more rational choices in accordance with economic decision theories, such as less temporal discounting [33], [34], [35], less loss aversion [33], [34], less framing effect and conjunction fallacy [36], and better performance in a sequential Prisoner’s Dilemma game [34], the present study suggested that people with higher cognitive abilities (intelligence and executive function) are more likely to engage the GF strategy. It should be emphasized that our subjects were chosen from a top-tier university in China, and they had an averaged intelligence of 124±9, as measured by both RAPM and WAIS. Yet they on average showed significant GF. More important, correlational analysis further suggested that the higher the cognitive ability they have, the more likely they engage the GF. There are two potential reasons for why this may occur. First, it is possible that the neural mechanisms of affective decision-making are weaker to begin with in those individuals. Another potential reason is that people with higher cognitive ability tend to exert stronger control over their affective/emotional systems thus rendering them inefficient in executing their functions.

Unlike the other biases where heuristic response was associated with strong emotional responses, the implementation of the GF strategy requires great cognitive control [4]. In contrast to the win-stay-loss-shift strategy that is likely to be guided by a model-free reinforcement learning mechanism, the GF strategy represents a case in which subjects showed a win-shift-loss-stay pattern, as guided by a false “world model”. To implement this counterintuitive decision strategy thus requires subjects to hold the prepotent WSLS response and switch to the opposite response. Consistently, the GF is associated with strong LPFC activation [4], [10]. Also in accordance with this view, it has been found that subjects engaged more GF when the inter-trial interval was increased, thus allowing more time and cognitive resources to implement the strategy [37].

On the other hand, a strong affective decision making mechanism could help create an affective label to a disadvantageous choice, e.g., the recently unrewarded color, and thus counteract this fallacy. This signal is particularly important to alarm the negative future consequences of a particular choice, and thus prevent subjects from further gambling after a series of losses. The importance of this alarm signal has been studied under the somatic marker framework, and patients who lose the capacity to trigger this signal (somatic marker) from prior reward/punishment begin to make maladaptive choices, despite maintaining a high level of intellect [38].

Consistent with many previous studies, the present large-sample study suggested that the IGT captures performance that is separated from the cognitive abilities, such as general intelligence, executive control and response inhibition. Our factorial analysis results are in line with a recent meta-analysis of 43 studies aimed at examining the relationship between IGT performance and cognitive ability, which concluded that the majority of the existing studies found non-significant correlations, and the minority of studies that reported statistically significant effects only revealed small to modest effect sizes [39]. Lesion study further showed that patients with lesions in the VMPFC were impaired on the IGT, but not on the working memory tasks. In contrast, patients with DLPFC lesions were impaired on the working memory tasks but not on the IGT [40]. Using cognitive and decision making tasks that tap into the functions of the lateral and medial PFC, our results suggest a differential role of the lateral and medial PFC in maladaptive decision making such as the GF. Future studies should examine whether the personality variables may also contribute to the GF, such as the rationality measured by the framing biases, the self-control, and the risk-taking attitude, which have been also associated with functions in the medial and lateral PFC. For example, the framing effect has been associated with activation in the VMPFC [41], the risk-taking is associated with activation in both the ventral and dorsal MPFC [42], and the self-control has been related to functions in the lateral prefrontal cortex [43]. Moreover, future studies should examine the relationship between the GF, and the anatomical, functional and genetic variances across individuals, which would provide further evidence for the genetic and neural mechanisms of the GF.

Taken together, emerging evidence has converged to suggest that the GF is contributed by three factors: (1) a false world model, (2) strong cognitive mechanisms, and (3) poor affective decision making mechanisms. The same three-component model can be applied to other types of belief-based or model-based decision-making (Figure 2). For example, when guided by a real world model that “vegetable is good for health”, a strong cognitive mechanism implemented by the lateral prefrontal cortex can exert self-control to modulate the affective decision mechanism and help to overcome the tendency for fat and sweet food [43]. The same lateral prefrontal cortex system, when being ‘hijacked’ by a false world model, like the GF, can also contribute to maladaptive decisions, whereas the affective decision making mechanism that help to differentiate good from bad choices. These results provide a novel insight into the mechanisms underlying adaptive and maladaptive decisions.
